# Papillary renal neoplasm with reversed polarity: A case report of a rare renal tumor

**DOI:** 10.1016/j.ijscr.2025.111517

**Published:** 2025-06-14

**Authors:** Swetha Kannan, Wania Mohammad Akram, Muhammad Elmussareh, Rana Saleh

**Affiliations:** a Final Year Medical Students, Gulf Medical University, United Arab Emirates; b Consultant Urological Surgeon, American Hospital Dubai, United Arab Emirates; c Specialist Anatomic Pathologist, American Hospital Dubai, United Arab Emirates

**Keywords:** Papillary neoplasm, Renal cell, Carcinoma

## Abstract

**Introduction and importance:**

Papillary Renal Neoplasm with Reversed Polarity (PRNRP) is a rare renal tumor first described in 2019. It is characterized by low-grade nuclei, eosinophilic cytoplasm, and an inverted nuclear arrangement. Unlike papillary renal cell carcinoma (PRCC), PRNRP frequently harbors KRAS mutations and lacks the typical trisomies of chromosomes 7 and 17. Histopathology and immunohistochemistry remain the gold standard for diagnosis.

**Case presentation:**

A 52-year-old woman with Type 2 Diabetes Mellitus, hypertension, and chronic kidney disease presented with worsening renal function. Imaging revealed a Bosniak type IV cyst in the right kidney, raising suspicion for malignancy. She underwent a robotic partial nephrectomy, and histopathology confirmed PRNRP (pT1a, WHO/ISUP grade 1). The tumor was completely resected, and no recurrence or metastasis was noted. PRNRP poses diagnostic challenges due to its rarity and overlap with other renal tumors. It is often discovered incidentally, with imaging offering limited preoperative specificity. Histopathologic criteria include thin papillary structures, interstitial vitrification, eosinophilic cytoplasm, and nuclear reversal. PRNRP follows an indolent course, with no reported cases of recurrence or metastasis post-resection.

**Clinical discussion:**

Papillary Renal Neoplasm of Reversed Polarity (PRNRP) is a rare variant of renal cell carcinoma characterized by its distinct histological feature of reversed polarity, where the epithelial cells exhibit basally located nuclei and apical cytoplasm. This uncommon tumor type poses diagnostic challenges due to its overlapping features with other renal neoplasms, underscoring the importance of histopathological examination for accurate identification and management.

**Conclusion:**

Current treatment parallels renal cell carcinoma management, primarily surgical excision, with surveillance recommended for long-term follow-up. PRNRP is a distinct renal neoplasm with an excellent prognosis. Further research is needed to refine diagnostic criteria, understand molecular mechanisms, and optimize treatment strategies. Increased awareness among clinicians and pathologists can aid in accurate diagnosis and management.

## Highlights

• Papillary Renal Neoplasm with Reversed Polarity (PRNRP) is a rare renal tumor first described in 2019.
• Current treatment parallels renal cell carcinoma management, primarily surgical excision and long term surveillance.
• Further research is needed to refine diagnostic criteria and optimize treatment strategies.
• Increased awareness among clinicians and pathologists can aid in accurate diagnosis and management.

## 1 Background

Papillary renal neoplasm of reversed polarity (PRNRP) is a rare tumor which was recently explained and documented in 2019 [4]. It exhibits a stark difference to papillary renal cell carcinoma (RCC) [1]. It is characterized by frequent KRAS mutations. PRNRP is a small, clearly defined, frequently encapsulated cystic tumor featuring loose papillary structures. The cuboidal tumor cells typically exhibit eosinophilic cytoplasm, with their nuclei positioned at the pole opposite the basement membrane, and they have a low nuclear grade according to the World Health Organization (WHO)/International Society of Urologic Pathologists (ISUP) classification. The fibrovascular cores may appear hyalinized or edematous. It is rare to observe macrophage clusters or intracellular hemosiderin, and psammoma bodies or necrosis should not be present [2]. In contrast, papillary renal cell carcinoma type 1 exhibits delicate papillae covered by a single layer of cells with scanty cytoplasm with nuclei generally located in a single layer on the basement membrane [3]. In terms of immunophenotype, this tumor is consistently positive for CK7 and GATA3, and negative for CD117 and vimentin. CD10 and AMACR may show weak and focal positivity. KRAS mutations are commonly present in PRNRP, although only 32 % of cases exhibit chromosomal abnormalities involving chromosomes 7, 17, and Y [2]. After complete resection, no instances of recurrence, metastasis, or tumor-related deaths have been reported.

## 2 Methodology

The work has been reported in line with the SCARE criteria [10].

## 3 Case

A 52-year-old woman with a history of Type 2 Diabetes Mellitus, dyslipidemia, and hypertension presented to the Nephrology clinic for evaluation of newly detected elevated serum creatinine of 111 μmol/L (increased from 66 μmol/L the previous year), non-nephrotic range proteinuria, and macroalbuminuria. Urinalysis also showed bacteriuria, which was treated with Fosfomycin 3 g given in three doses. She had no urinary symptoms. Her medical history is significant for recurrent lower urinary tract infections and a laparoscopic ovariectomy. She is a known case of chronic kidney disease with a GFR of 50. Her regular medications include ezetimibe/rosuvastatin 10 mg, subcutaneous insulin aspart and degludec, and perindopril 5 mg.

Ultrasound of the kidneys and bladder showed a right interpolar hypoechoic cyst with a central echogenic nodule and a stable upper pole cyst in the left kidney. MRI of the kidneys (with and without contrast), renal function panel, blood glucose, serology for glomerular diseases, urinalysis, and culture were ordered. The patient was advised to maintain a blood pressure log and to continue blood glucose monitoring in coordination with her endocrinologist. Serologic workup for glomerular diseases was unremarkable.

MRI [4] showed a Bosniak type IV cyst in the right kidney and a Bosniak type II hemorrhagic cyst in the left kidney. She was advised to seek immediate evaluation in the Urology clinic based on these findings. She was also referred to Gynecology for consideration of topical estrogen cream due to persistent bacteriuria. She was advised to avoid nephrotoxic medications such as NSAIDs.

Her case was reviewed at the multidisciplinary tumor board meeting. The MRI of the abdomen and pelvis showed a 3.2 × 3.6 × 3.7 cm cortical, exophytic mass in the interpolar region, with low signal intensity on T2 and an internal irregular nodule with high signal intensity on T1, showing reversal of signal intensity. She was reviewed in the Urology clinic and informed that the majority of Bosniak type IV cysts (approximately 90 %) are malignant. The option of undergoing a robotic partial nephrectomy was discussed, and she was counseled on the risks and benefits of surgery. A CT chest was ordered to complete staging and was unremarkable.

She successfully underwent a robotic-assisted laparoscopic partial nephrectomy, and the cyst [1] was fully excised. Surgical pathology showed a **Papillary Renal Neoplasm with Reversed Polarity** in the interpolar region, measuring 2.8 cm. The tumor was staged as pT1a, WHO/ISUP grade 1, with no evidence of necrosis, sarcomatoid, or rhabdoid features. Surgical margins were negative for malignancy.

Figs. 2a, 2b, and 2c show the histopathological analysis of the specimen. IHC panel is shown in Fig. 3.

**Fig. 1:**
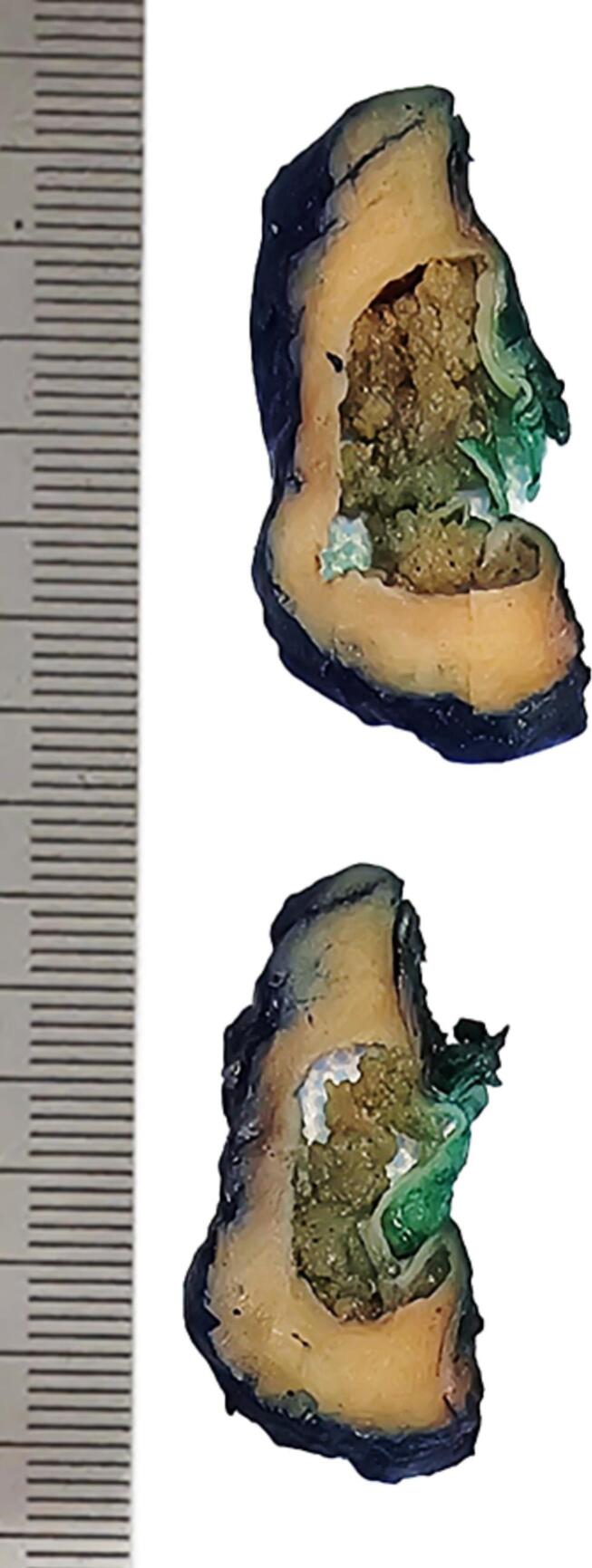
Gross specimen of partial nephrectomy specimens shows involvement by cystic neoplasm with internal papillary growth.

**Fig. 2:**
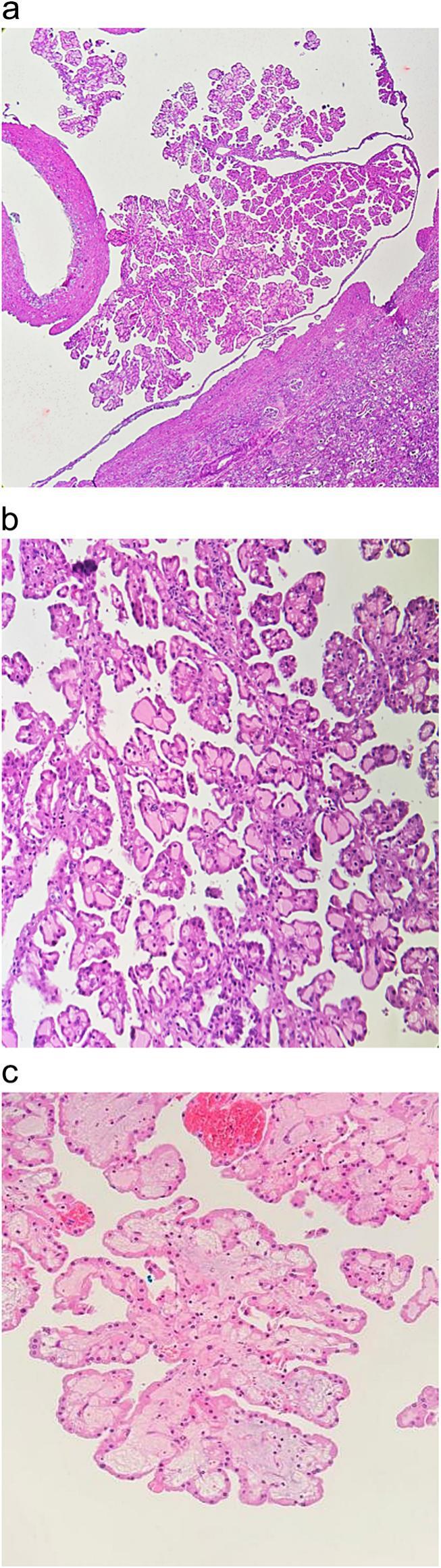
**(a): H&E low power:** Nests of Oncocytic tumor cells with uniform nuclei and abundant Eosinophilic cytoplasm. **(b)H&E medium power:** The papillae are composed of thin fibrovascular cores lined by a single layer of low cuboidal cells. **(c): H&E high power:** The lining epithelial cells have eosinophilic cytoplasm with small bland uniform nuclei located at the apical cell border away from the basement membrane.

**Fig. 3:**
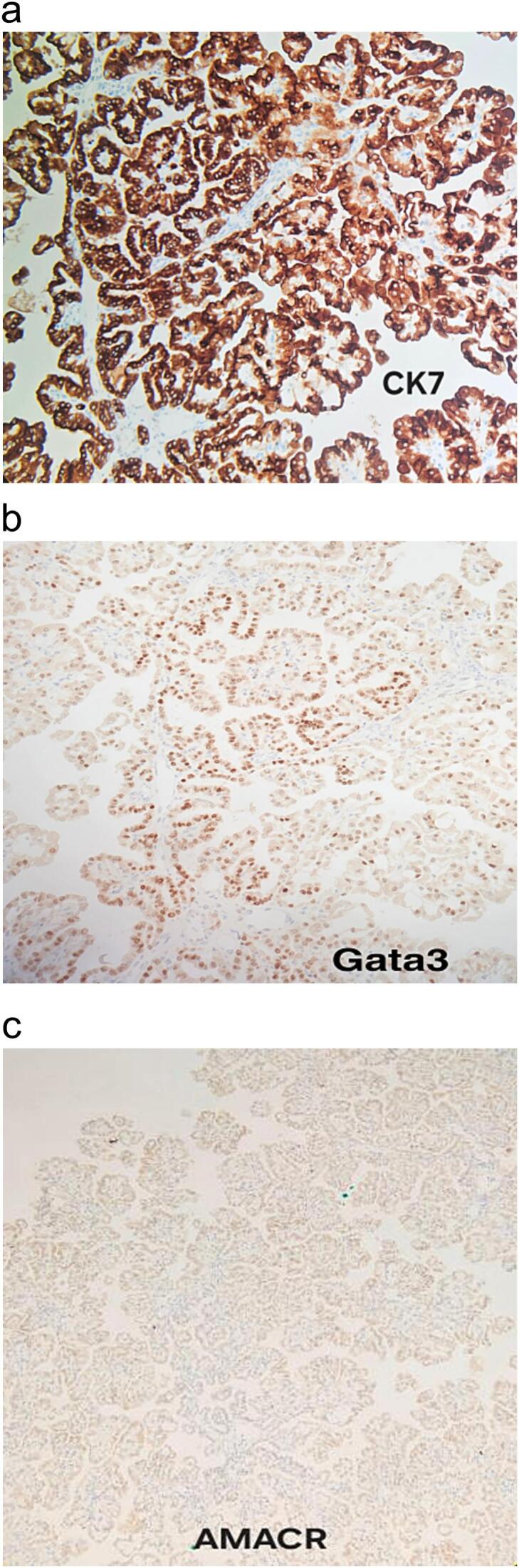
IHC panel: Tumor cells are diffusely positive for CK7 (3a), and Gata3 (3b), AMACR (3c) stain is weak.

**Fig. 4:**
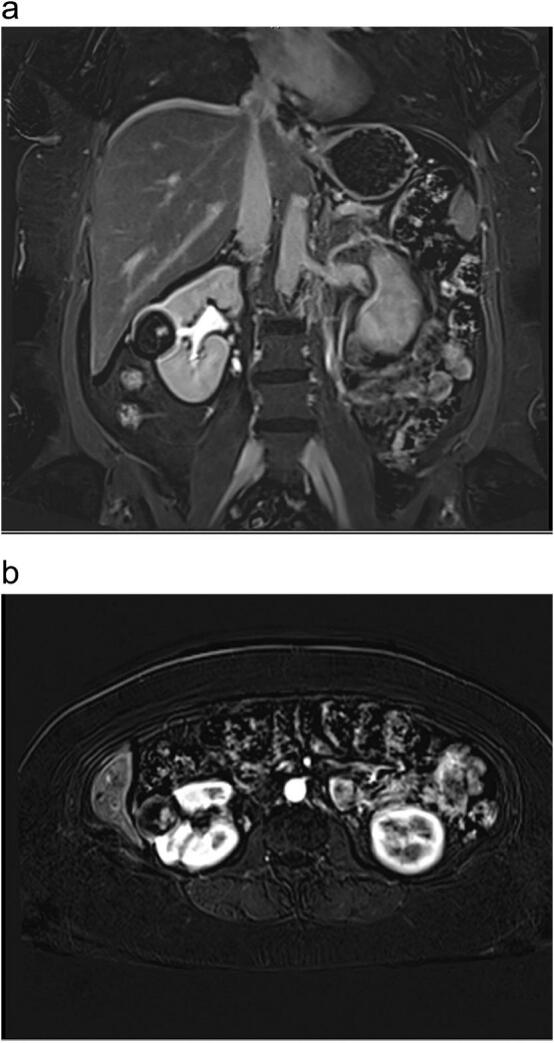
**(a) and (b):** MRI abdomen and pelvis showed interpolar region cortical exophytic 3.2 × 3.6 × 3.7 cm mass demonstrating low signal intensity on T2, with internal irregular nodule of high signal intensity on T1. There is reversal of signal intensity.

The patient made a full recovery with no postoperative change in renal function. She was informed that the tumor is indolent and carries a good prognosis. Regular follow-up was advised (see Fig. 1, Fig. 2, Fig. 3, Fig. 4).

## 4 Discussion

Papillary Renal Neoplasm of Reversed Polarity (PRNRP) is a rare variant of renal cell carcinoma characterized by its distinct histological feature of reversed polarity, where the epithelial cells exhibit basally located nuclei and apical cytoplasm. This uncommon tumor type poses diagnostic challenges due to its overlapping features with other renal neoplasms, underscoring the importance of histopathological examination for accurate identification and management.

PRNRPs are typically asymptomatic and are often found incidentally during imaging presenting significant challenges for preoperative diagnosis Although most PRNRPs are small, as they grow, they can exert pressure on nearby organs, affect kidney function, and potentially lead to complications such as bleeding or rupture. Imaging studies offer limited diagnostic value because of the rarity of the condition. Histopathology and immunohistochemistry remain the gold standard for diagnosing PRNRP [3].

Chang et al. [5] proposed the following four diagnostic criteria for PRNRP:

(I) Predominantly protruding thin papillary or tubular papillary growth;
(II) Focal or diffuse interstitial vitrification;
(III) Eosinophilic fine granular cytoplasm;
(IV) Tumor nuclei are neatly arranged at the apex of the cytoplasm, away from the basement membrane, exhibiting “reverse polarity” characteristics, with uniform size and low nuclear grade.

In a systematic literature review done in a case report published, 11 publications describing 97 cases were finally identified. The review series included 97 patients (56 men and 41 women) with a definite diagnosis of papillary renal tumor with reverse polarity. The evaluation showed that 31 cases of PRNRP occurred in the left kidney, and 43 cases occurred in the right kidney. The age of PRNRP patients ranged from 35 to 82 years. The diameter of PRNRP ranged from 0.8 to 8.5 cm, with an average diameter of 2.1 cm. The World Health Organization (WHO)/International Society of Urological Pathology (ISUP) showed low nuclear grade, and most of the reported PRNRP cases were staged as pT1 [4].

The inverted nuclear polarity feature has been described in earlier literature but received limited attention until recent studies. Lefèvre et al. [6] studied 10 cases of oncocytic renal papillary tumors, showing no trisomy 7 or 17 and suggesting a distinct variant of PRCC. Kunju et al. [7] identified seven papillary renal tumors with unique features, including oncocytic cytoplasm and low-grade nuclei, which had no recurrence or metastasis. Park et al. [8] described oncocytic papillary renal cell carcinoma with an inverted nuclear pattern, sharing similarities with type 1 PRCC and an indolent course. Saleeb et al. [9] proposed a new PRCC subtype with improved clinical classification, while Al-Obaidy et al. [3] analyzed 18 cases of papillary renal neoplasm with reverse polarity, emphasizing favorable prognosis and lack of recurrence.

The treatment of papillary renal neoplasm with reversed polarity (PRNRP) is still evolving due to its rarity, but it generally follows the treatment guidelines for renal cell carcinoma (RCC), especially type 1 PRCC. For localized tumors, nephrectomy is performed based on tumor size and location. Advanced or metastatic cases may be treated with targeted therapies like sunitinib or immune checkpoint inhibitors like nivolumab. For small, low-grade tumors, active surveillance with periodic imaging is an option. Radiotherapy and molecular profiling may be considered in rare or advanced cases. Regular follow-up is essential to monitor for recurrence or metastasis.

The prognosis of papillary renal neoplasm with reversed polarity (PRNRP) is generally favorable due to its indolent nature. Most patients present with small, low-grade, localized tumors (stage pT1), and surgical resection is often curative. Recurrence or metastasis is rare. Chromosomal abnormalities like trisomies of chromosomes 7 and 17 are present but don't affect the overall prognosis. With regular monitoring, patients typically have long-term survival, similar to type 1 papillary renal cell carcinoma.

## 5 Conclusion

In conclusion, papillary renal neoplasm with reversed polarity (PRNRP) is a rare and distinct subtype of renal tumor that presents unique diagnostic and treatment challenges. Although it generally has a favorable prognosis, its rarity highlights the need for further research to improve our understanding and management of this rare cancer. More studies are required to refine diagnostic criteria, treatment protocols, and explore its molecular mechanisms. Current management often follows guidelines for renal cell carcinoma, but tailored approaches based on PRNRP's unique characteristics could enhance patient outcomes. Long-term studies will be essential to assess the effectiveness of targeted therapies and refine surveillance strategies.

## Author contribution

study concept or design- Dr. Muhammad Elmussareh and Dr.Rana Saleh

data collection- Swetha Kannan and Wania Akram

data analysis or interpretation- Swetha Kannan and Wania Akram

writing the paper- Swetha Kannan and Wania Akram

## Consent

Written informed consent was obtained from the patient for publication and any accompanying images. A copy of the written consent is available for review by the Editor-in-Chief of this journal on request.

## Ethical approval

Ethical approval was exempt by the supervisor physician as he took informed consent from the patient. And the patient's identity is not disclosed in the case report. This is in accordance with the hospital protocol.

## Guarantor

Dr. Muhammad Elmussareh.

## Source of funding

None.

## Declaration of competing interest

None.
